# Pediatric chronic kidney disease rates in Southern Israel are higher than reported

**DOI:** 10.12688/f1000research.2-186.v1

**Published:** 2013-09-13

**Authors:** Daniel Landau, Ruth Schreiber, Anya Kleinman, Alina Vodonos, Hannah Shalev

**Affiliations:** 1 Pediatric Nephrology, Soroka University Medical Center, Ben Gurion University, Beer Sheva, Israel; 2 Clinical Research Center, Soroka University Medical Center, Ben Gurion University, Beer Sheva, Israel

## Abstract

**Background**: The incidence and prevalence of pediatric chronic kidney disease (p-CKD) in developed countries has previously been estimated to be 12 and 75 cases/10 ^6^ population respectively, much lower than reports in young adults (age 20-40) (40,000/10 ^6^). Thus, the extent of p-CKD may be underestimated.

**Methods:** Being the only Pediatric Nephrology center in Southern Israel, we reviewed all detected cases of p-CKD (stages 1-5) between 1994-2008. We then prospectively summarized the incidence and prevalence of CKD between 2009-2010.

**Results: **We retrospectively identified 192 children (53.9% of Bedouin origin, 53.4% males, median diagnosis age: 1 year) with CKD. The prevalence in December 2008 was 795 cases/10 ^6^ for all CKD stages and 331/10 ^6^ for CKD stage >2. Calculated incidence for the study period (1994-2008) was 46/10 ^6^/year. The main CKD etiologies were: hypodysplasia: 35%; obstructive uropathy: 13%; genetic renal diseases: 28% and glomerulonephritis: 15%. The proportions of children in each CKD stage were as follows: stage 1: 50%; stages 2-4: 30%; stage 5: 20%. During a subsequent two-year study period we identified 26 new CKD cases (incidence: 54 cases/10 ^6^/year).

**Conclusions:** p-CKD rates in our area are higher than reported and maybe even higher if asymptomatic populations are screened. Fifty percent of detected cases have CKD stage 1. This may contribute significantly to CKD beyond the pediatric age.

Box 1. **Key Notes.**

- In chronic kidney disease (CKD) there is permanent bilateral kidney damage, structural or functional, with or without a decrease in glomerular filtration rate. Reported childhood CKD rates are far lower than that of young adults and depend on discovered cases, possibly missing asymptomatic cases.
- In our population-based study, we found pediatric CKD rates 10 times higher than previous studies, 50% of them CKD stage 1. This may explain the CKD burden beyond pediatric age.

## Introduction

Chronic kidney disease (CKD) is defined as evidence for bilateral kidney damage for more than 3 months, which can be structural or functional, with or without a decrease in glomerular filtration rate ^1^. The presence of CKD at any stage is a strong risk factor for renal function deterioration during lifetime, many times beyond the pediatric age ^2^.

The prevalence of pediatric CKD (p-CKD; stage ≥2) in developed countries (such as in Italian ^3^ and Spanish studies ^4^) has been estimated to be as high as 75 cases/10 ^6^ population at risk. However, until recently, such estimates were based on CKD retrospectively detected by tertiary care centers, which does not allow the determination of true incidence as patients who do not have advanced CKD ("overt CKD") may not be identified. In addition, these pediatric numbers are much lower than reports from American young adults (up to 40,000/10 ^6^ for ages 20–40 by the National Health and Nutrition Examination Survey (NHANES studies) ^5^. Thus, the extent of p-CKD may be underestimated.

The Negev population receives tertiary care services through a single medical institution, the Soroka University Medical Center (SUMC), which serves a population (including referrals) of 300,000 children and takes care of more than 15,000 deliveries a year. The SUMC Pediatric Nephrology service is the only caregiver for children in the area affected by kidney and urinary tract diseases. The population served by this hospital is composed of two major subpopulations, usually living in separate settlements: Jews (75%) and Arab-Bedouins (25%). The consanguinity rate among Jewish couples is nowadays low and estimated as 2.3%, including 0.8% first cousin marriages ^6^. The Negev Bedouins' current estimated population is 200,000. The total fertility rate in the Bedouin-Muslim population in 2006 was 7.3, compared with to 3.3–4.1 in other Muslim populations in Israel and 2.62 in the Jewish population in southern Israel. In Bedouin society, cousin marriage is the preferred type of marital union and traditionally involves marriage between first-degree cousins ^7, 8^. Currently, 60% of this population is under 19-years-of-age ^9^. More than half of the Bedouin population live in unrecognized villages and shanty towns, and suffer from a high rate of unemployment. However, all Israelis are entitled to medical care as a result of a National Health Insurance plan introduced in 1995. The reported pediatric CKD stage 5 end-stage renal disease (ESRD) incidence for 2006 in this area was 19 cases/10 ^6^/year ^10^, similar to reports from developed countries ^11^. Previous studies from this center provide reliable population-based epidemiological data ^12, 13^. The purpose of this study was to assess the prevalence and etiology of p-CKD in Southern Israel.

## Material and methods

The study protocol was approved by the hospital's ethics committee (approval number: 10568). We reviewed all recorded cases of p-CKD among inhabitants of the Negev area (and not referrals from other country areas) during the January 1994-December 2008 period, based on a review of medical records of children detected by the Pediatric Nephrology service to have this condition. This was double-checked by screening the hospital's Medical Records department discharge diagnoses to look for additional children (age 0–19 years) with CKD diagnosis. For the retrospective analysis, we graded based on the worst CKD stage reached by the children. We calculated CKD prevalence and stage according to the living child’s status (CKD stage 1–5) as at Dec 31, 2008. In addition, we prospectively collected all new CKD cases diagnosed in our hospital, from January 1, 2009 until December 31, 2010. Data from medical records were entered into standardized forms, including demographics, age of onset, underlying renal disease, etc. The standardized form used is provided in the data file.

### Definitions

Estimated glomerular filtration rate (eGFR) was calculated by the original Schwartz formula ^14^. Hypodysplasia was defined by imaging studies as a congenital reduced kidney size and maldevelopment of the renal tissue (including renal scars), with or without associated malformations in the urinary tract (hydronephrosis, hydroureter, vesicoureteric reflux), in both urinary tract systems. Unilateral urinary tract anomalies and/or a single kidney were not included unless an additional feature of CKD (such as proteinuria or hypertension) was present. Obstructive uropathy was divided between neurogenic bladder and congenital obstruction. Genetic renal disease was diagnosed only for monogenic diseases that are known to be associated with a decreased renal function with age. Therefore, diseases such as Bartter syndrome or nephrogenic diabetes insipidus were not included. CKD stage 1 was defined as an eGFR of equal or more than 90 ml/min/1.73 m ^2^ (a normal GFR value) but with the presence of either persistent albuminuria (as may exist for reflux or diabetic nephropathy) or bilateral structural renal anomalies (as in polycystic kidney disease, hypodysplasia, or bilateral renal scars). CKD stage 2 was defined as a eGFR between 60–90 ml/min/1.73 m ^2^; CKD stage 3 was a eGFR between 30–60 ml/min/1.73 m ^2^, CKD stage 4 was a eGFR between 15–30 ml/min/1.73 m ^2^ and CKD stage 5 was defined as a GFR of < 15 ml/min/1.73 m ^2^.

### Statistical analysis

A direct age adjustment method was performed to compare the prevalence and incidence of p-CKD between the Jewish and Bedouin populations using children under the age of 19 as standard population. Population statistics were obtained from The Israeli Central Bureau of Statistics national census. Statistical analysis was performed using the SPSS software, version 18.

## Results

**Figure 1.**
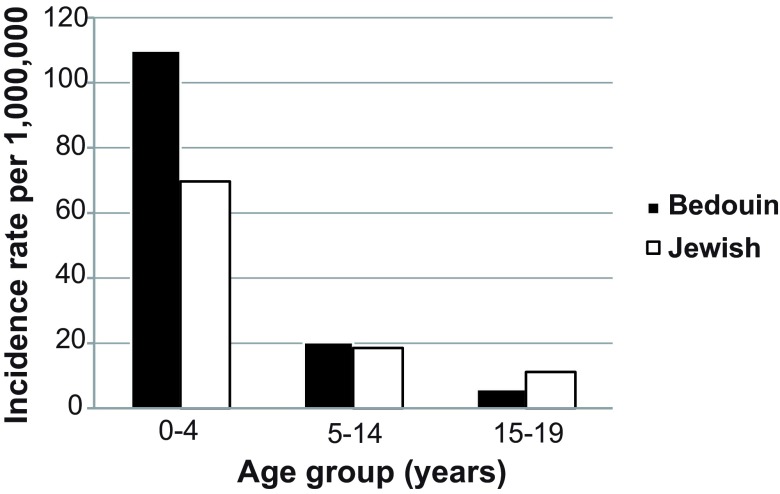
Y axis; age adjusted annual chronic kidney disease (CKD) incidence rate (per million). X axis: age groups (years). Black bars: children of Bedouin origin. White bars: children of Jewish origin. No p values for comparison are provided since the total population (and not a sample) is analyzed.

We identified 192 living p-CKD patients at December 31, 2008 (46.1% of Jewish origin, 53.4% males) during the retrospective study period. The population at risk at that time was 241,400, as provided by the Israeli Census Bureau ( http://www1.cbs.gov.il/reader/shnaton/templ_shnaton_e.html?num_tab=st02_10x&CYear=2010), providing a point prevalence of 795.4 (95% confidence interval (CI): 690.6–916.0) per million ( Table 1). The age-adjusted Jewish p-CKD prevalence rate was 628.2 (95% CI: 549.2–751.9) per million, whereas the age-adjusted Bedouin p-CKD prevalence rate was 850.2 (95% CI: 764.4–1000.8) per million. Incidence for the whole study period (1989–2009) was 45.4 (95% CI: 39.7–53.0) per million. When different pediatric age groups were analyzed separately, a higher rate was found in younger children (age 0–4 yrs) of Bedouin origin than children of Jewish origin. Rates were similar between the two groups at ages 5–14 and 15–19 years ( Figure 1).

**Table 1.** Southern Israel pediatric chronic kidney disease (CKD stages 1–5), based on the retrospective analysis. * Refers to living patients aged < 19 years on December 2008. Prevalence is adjusted for age. ** Mostly autosomal dominant polycystic kidney disease (ADPKD); *** Mostly atypical hemolytic uremic syndrome and infantile nephronophtisis. No p values for comparison are provided since the total population (and not a sample) is analyzed.

Measure	Total	Jewish	Bedouin
CKD cases*	192	89 (46.1%)	103 (53.4)
Population at risk (*1,000)	241.4	135.5	105.9
Prevalence (cases/10 ^6^)*	795.4	642.6	875.7
Mean ± SD diagnosis age (yr)	4.9±6	6.3±6.4	3.6±5.4
Median diagnosis age (yr)	1	4.0	0.3
% male	58	57	59
CKD stage (%)			
1	50.3	53	47
2	16.5	17	16
3	7.3	5	9
4	5.8	9	4
5	19.6	15	23
CKD etiology (%)			
Hypodysplasia	35	33	36
Obstructive uropathy (neurogenic bladder)	12.7 (6.2)	8 (1.6)	17 (7.3)
Genetic renal disease	28.1	29**	28***
Glomerulonephritis	14.6	19	11
Other	9.6	11	7
Death (%)	4.6	0.8	8
Transplant (%)	14.2	13	15.2

The mean age of diagnosis was 4.9±6 years, but the median age of diagnosis was 1 year. The main p-CKD etiologies were: hypodysplasia: 35%; obstructive uropathy: 13%; genetic renal diseases: 28% and glomerulonephritis: 15%.

The proportions of children in each CKD stage were as follows: stage 1: 50%; stages 2–4: 30%; stage 5: 20%. One hundred thirty-one children were diagnosed with CKD stage 1, which included 44 children with bilateral renal hypodysplasia (with or without renal scars), 10 children with obstructive uropathy (five of them with congenital obstruction of both kidneys and five with neurogenic bladder and bilateral renal scars), 35 children with genetic renal diseases (14 with autosomal dominant polycystic kidney disease (ADPKD) and 10 with the autosomal recessive form (ARPKD)), and 28 children with chronic glomerulonephritis (10 with IgA nephropathy, five children with Henoch Schoenlein purpura nephropathy and residual proteinuria). Miscellaneous diagnoses ^14^ included seven children and adolescents with diabetic nephropathy.

Children of Bedouin origin had a lower median age of diagnosis (0.3 vs 4 years in Jews, p < 0.01). Twelve children died during the retrospective study period, 11 of them of Bedouin origin, six of them reached ESRD due to two genetic renal diseases: atypical hemolytic uremic syndrome and infantile nephronophtisis. The other five children had hypodysplasia, four of them as part of multiple congenital anomalies, and only one of these 5 children reached ESRD. The one child of Jewish origin that died had Lowe syndrome and died of neurologic complications at CKD stage 2. The same rate of Jewish and Bedouin children (15%) had a renal transplant.

The prevalence of CKD at stage 2 or above was 331/10 ^6^ (95% CI: 266.3–412.4), a higher number than that reported from the series in Spain and Italy 71/10 ^6^ and 75/10 ^6^ ( Table 2). The male: female ratio and age of diagnosis were similar between the series. However, the fractions of CKD children who had a genetic renal disease or obstructive uropathy were higher in our area.

**Table 2.** Chronic kidney disease (CKD) stage 2–5: comparison with other epidemiological studies. * Age at registration: 6.9±5.4. ** Including congenital obstructive uropathy.

Measure	Southern Israel	Spain	Italy
Reference	This study	Areses *et al.* ^4^	Ardissino *et al.* ^3^
Date of evaluation	Dec 31, 2008	Dec 31, 2008	Jan 1, 2001
Age for inclusion (yr)	< 19	< 18	< 20
Population at risk (10 ^6^)	0.24	11.3	16.8
Prevalent cases	80	605	1197
Prevalence (cases/10 ^6^)	331.4	71	75
% male	72	66	66
Age of diagnosis (yr)	2.7±4.8	3.9±5	NA*
CKD etiology (%)			
Hypodysplasia	52.8	59	58**
Genetic renal disease	28	14	15.6
Obstructive uropathy	10.1	NA	3.7
Glomerulonephritis	4.5	3	5.8
CKD stage (%)			
2	50	42	39
3	21.2	40	35
4	15	15	26
5	13.8	4	Not included

During a subsequent 2 year period (January 2009 – December 2010), we identified 26 new children with CKD, providing a yearly incidence of 53.8 (95% CI: 31.5–92.1) new cases/10 ^6^/yr (half of them at CKD stage 2 or above). The mean age of diagnosis for CKD (stage 2 or above) in the prospective cohort was 2.7±4.8 years (interquartile range: 0–6 yrs, median age: 0.5 yrs).

Pediatric chronic kidney disease rates in Southern Israel 1) Data acquisition form2) CKD Database raw data. See materials and methods in the main paper for definitions. Upper row abbreviations: DOB: date of birth; B/J: Bedouin or Jewish origin; H/GRD/GN/OU/O: hypodysplasia/ genetic renal disease/ glomerulonephritis/ obstructive uropathy/ other; NB or CU: neurogenic bladder or congenital obstructive uropathy; HTN: hypertension; EPO Rx: erythropoietin therapy

## Discussion

The p-CKD prevalence reported in this study is higher than that reported for other recent series from developed countries, such as Italy and Spain ( Table 2). We chose to compare our data with these two European countries because of the similarities in accessibility to medical services. A Universal Health Care system by law has been implemented in Israel since 1995 and provides comprehensive medical care to all Israelis. Infant mortality has dropped in Israel in the past decades and is now comparable to most developed countries ^15^. In addition, access to prenatal diagnosis by different screening tests including frequent fetal ultrasound studies has become the standard of care for the great majority of the Israeli population: 90% of Israeli pregnant women have at least one fetal ultrasound performed (Drs Shochat and Romano-Zelicha, Israeli Ministry of Health survey, personal communication). This may allow the detection of many congenital anomalies of the kidneys and urinary tract as well as some of the genetic renal diseases that cause CKD (such as polycystic kidney disease). It may also be the reason for the low median age of detection of p-CKD in our cohort (1 year for the general cohort and as low as three months for the Bedouin population) ( Table 1). The lower mean age of diagnosis for the Bedouin population may reflect the higher rate of genetic renal diseases in our area (28% in Southern Israel vs. 14% and 15.6% in Spain and Italy respectively) ^11^. In addition, we found a higher rate of obstructive uropathy due to neurogenic bladder, most of these cases in the Bedouin population, similar to reports from Turkey ^16^, again raising the possibility of the contribution of consanguinity or poverty (such as lower access to folic acid supplementation). However, a similar male predominance and high prevalence of renal hypodysplasia as the main etiology for CKD is seen in these three series.

It must be remembered that this apparent higher p-CKD rate is still an underestimation of the probable true p-CKD prevalence that may be detected if population screening studies are performed. Such studies have been performed in recent years in Australia, where persistent albuminuria or hypertension were found in 1–2% of prospectively screened school-age children ^17^. Similar reports come from Japan, Taiwan and Korea ^18^. Furthermore, the striking difference in CKD rates between pediatric and young adult populations needs to be addressed. The overall prevalence of CKD stages 1 to 4 in adults increased significantly in the 1999 to 2004 period compared with 1988 to 1994 (13.1 versus 10.0 percent respectively) ^19^. Data from the NHANES 2003–2006 survey, with GFR estimated by the CKD-Epidemiology Collaboration study (CKD-EPI) equation, showed that the overall prevalence of CKD stages 1 through 5 among adults is 14.2% ^20^. In other countries, CKD prevalence ranges between 1 to 30% ^21^. This is not only true for the aged population, since a CKD (stage ≥2) prevalence rate as high as 4% (or 40,000/10 ^6^ has been reported for the 20–40 year old population ^22^.

There are fewer reports on the incidence of CKD among adults. In the Framingham cohort (n = 2580), after a mean follow-up of 18.5 years, 244 participants (9.4%) had developed CKD stage 3 and above ^23^, which is a yearly incidence of about 500 new patients/million/year. If one assumes an overall CKD prevalence of 10% (or 100,000 per million) this provides a prevalence-to-incidence ratio of 200 in adults, which is expected given the chronic nature of CKD.

A major cause of mortality in adult CKD is increased cardiovascular comorbidity, in addition to ESRD progression ^24^. When compared to the pediatric population (which includes the first two decades of life vs. the subsequent average of six decades in adults), where no clinical cardiovascular morbidity has yet developed and mortality is lower than adults, one would expect a higher than reported prevalence-to-incidence ratio, which has been only around 6:1 in various previous studies. In our study, we report a ratio of around 15:1. This suggests an underdiagnosis of CKD in the pediatric population. In addition, all studies performed to-date exclude CKD stage 1, which may be incorrect given the more long-term follow-up required for pediatric patients. The potential for gradual clinical progression, even from CKD stage 1, has been demonstrated for renal hypodysplasia in Italian ^25^ and Dutch ^26^ surveys, and is a well recognized requirement for diabetic nephropathy, in both type I ^27^ and, more importantly, type II diabetes mellitus ^28^. Supporting all these observations is a recent series of publications based on a true population frequency in Turkey, which demonstrate that the prevalence of childhood CKD approaches 1%, a number in the same order of magnitude as that of the younger adult cohort in the United States ^29^.

In summary, we report on a higher prevalence and incidence for p-CKD in Southern Israel and propose that, if proper population-based screening studies are performed, then higher p-CKD values should be found in other developed countries. This may expand the contribution of pediatric renal disease to CKD even beyond the pediatric age.
