# Supplementary material for: Pediatric chronic kidney disease rates in Southern Israel are higher than reported
Source: F1000Res. 2013 Sep 13;2:186. [Version 1] doi: 10.12688/f1000research.2-186.v1 (PMC3829122; doi:10.12688/f1000research.2-186.v1)
Supplement: Pediatric chronic kidney disease rates in Southern Israel — 1) Data acquisition form2) CKD Database raw data. See materials and methods in the main paper for definitions. Upper row abbreviations: DOB: date of birth; B/J: Bedouin or Jewish origin; H/GRD/GN/OU/O: hypodysplasia/ genetic renal disease/ glomerulonephritis/ obstructive uropathy/ other; NB or CU: neurogenic bladder or congenital obstructive uropathy; HTN: hypertension; EPO Rx: erythropoietin therapy [file f1000research-2-1887-s0000.tgz › CKD_Database_form.pdf]

CKD Database for all Negev CKD children (age < 19 yrs): 1/1994- 12/2008

Soroka University Medical Center

PI: Daniel Landau, MD

Study # _____ Family Name: ____________ First Name: _________

ID#: __________________ DOB (mm/yyyy): _________

M/F: ___ B/J: ____ City: ______________

Current CKD Stage (1-5): ____

Hypodysplasia/GRD/GN/Obstr-Uropath/Other: ____

Family Hx of CKD (y/n): ________________

Renal Dx for CKD: ______________

If OU: **N**eurogenic **B**ladder or **C**ongenital **U**ropathy?: _______

Non renal Dx 2: _____________

Non renal Dx 3: ____________

Non renal Dx 4: _____________

Biopsy for Dx confirmation (y/n): _______

Age of Dx of CKD (yrs): ____________

Exitus (y/n): ____

Negev + CKD+Alive+Age < 19@ 12/08 (y/n): _______

HTN (y/n): ___

Acidosis (HCO3 Rx) (y/n): ___

EPO Rx (y/n): ___

Urologic surgery (y/n): _______

Transplanted (y/n): ____

Dialysis (y/n): ______

If yes PD=1; Hemo= 2 ; PD & hemo= 3: ___

U-Prot/Creat (mg/ mg) > 0.2 (y/n): ___

U-(alb/Cr) (mg/g) > 30 (y/n): ____
