# Supplementary material for: Pediatric chronic kidney disease rates in Southern Israel are higher than reported
Source: F1000Res. 2013 Sep 13;2:186. [Version 1] doi: 10.12688/f1000research.2-186.v1 (PMC3829122; doi:10.12688/f1000research.2-186.v1)
Supplement: Peer review history

# Peer review: Referee response for version 1

**Published:** 2013-10-03

**Authors:** Uri Alon

**Affiliations:** 1 Pediatric Nephrology, Children's Mercy Hospitals and Clinics, Kansas City, MO, USA

A note about the possible reason(s) for the high incidences like consanguinity and poverty should be added to the abstract's conclusion for completion, otherwise I approve this study.

I have read this submission. I believe that I have an appropriate level of expertise to confirm that it is of an acceptable scientific standard.

# Peer review: Referee response for version 1

**Published:** 2013-09-16

**Authors:** Craig Langman

**Affiliations:** 1 Department of Kidney Diseases (Nephrology), Chicago Children's Memorial Hospital, Chicago, IL, USA

What shall we learn from this report by Landau and colleagues from southern Israel? It is clear that when one looks at the incidence of CKD and ESRD in the United States in young adults (<39 years), there has been tremendous increase of theses diseases over the past two decades.  In part, this is due to paediatric CKD manifesting itself in young adulthood due to loss of nephrons from the very conditions uncovered by the Landau group. Certainly with survival of very premature infants deprived of adequate in utero nephron development, and loss of nephrons for a multitude of reasons in neonates and older children (including acute kidney injury, multiple nephrotoxic medications, and chronic diseases that lead to increased patient survival but at the expense of nephron health) we have a true epidemic. This report goes a long way to beginning to warn paediatricians world-wide that rates of paediatric CKD cannot be left to those referral centers alone, but should be sought for in general visits to the paediatricians’ office with a careful history and physical examination followed by a consideration for testing that might include a kidney ultrasound to look for hypo-dysplasia, and performance of a test for low-grade albuminuria (termed microalbuminuria) as early signs. Identification of such early disease (CKD1-2) may lead to useful strategies to preserve functional nephron mass as long as possible. The current report provides excellent evidence in this regard.

I have read this submission. I believe that I have an appropriate level of expertise to confirm that it is of an acceptable scientific standard.
